# The sound-induced flash illusion reveals dissociable age-related effects in multisensory integration

**DOI:** 10.3389/fnagi.2014.00250

**Published:** 2014-09-24

**Authors:** David P. McGovern, Eugenie Roudaia, John Stapleton, T. Martin McGinnity, Fiona N. Newell

**Affiliations:** ^1^Trinity College Institute of Neuroscience, Trinity College DublinCollege Green, Dublin, Ireland; ^2^Intelligent Systems Research Centre, University of UlsterLondonderry, UK

**Keywords:** multisensory integration, sound-induced flash illusion, perception, time window of integration, aging

## Abstract

While aging can lead to significant declines in perceptual and cognitive function, the effects of age on multisensory integration, the process in which the brain combines information across the senses, are less clear. Recent reports suggest that older adults are susceptible to the sound-induced flash illusion (Shams et al., 2000) across a much wider range of temporal asynchronies than younger adults (Setti et al., 2011). To assess whether this cost for multisensory integration is a general phenomenon of combining asynchronous audiovisual input, we compared the time courses of two variants of the sound-induced flash illusion in young and older adults: the *fission* illusion, where one flash accompanied by two beeps appears as two flashes, and the *fusion* illusion, where two flashes accompanied by one beep appear as one flash. Twenty-five younger (18–30 years) and older (65+ years) adults were required to report whether they perceived one or two flashes, whilst ignoring irrelevant auditory beeps, in bimodal trials where auditory and visual stimuli were separated by one of six stimulus onset asynchronies (SOAs). There was a marked difference in the pattern of results for the two variants of the illusion. In conditions known to produce the fission illusion, older adults were significantly more susceptible to the illusion at longer SOAs compared to younger participants. In contrast, the performance of the younger and older groups was almost identical in conditions known to produce the fusion illusion. This surprising difference between sound-induced fission and fusion in older adults suggests dissociable age-related effects in multisensory integration, consistent with the idea that these illusions are mediated by distinct neural mechanisms.

## Introduction

The aging process is accompanied by a gradual decline in many aspects of perceptual function. Perhaps the most salient examples of sensory decline are found in vision and audition, where visual acuity and auditory sensitivity decrease in an age-dependent manner (Pitts, 1982; Liu and Yan, 2007). More recent work has examined whether aging affects the way in which information is combined across the senses, and whether this multisensory integration could help to compensate for the reduced sensitivity to unisensory information. So far, however, the evidence for an age-related benefit of multisensory integration remains surprisingly equivocal with examples of multisensory enhancement (Laurienti et al., 2006; Peiffer et al., 2007; Diederich et al., 2008) and impairment (Setti et al., 2011; Stapleton et al., 2014), as well as instances where integration appears to be reduced in older adults (Stephen et al., 2010; Roudaia et al., 2013). As such, it remains an open question as to whether multisensory integration generally compensates for age-related unisensory deficits or whether specific aspects of this integration process also decline with age.

**Figure 1:**
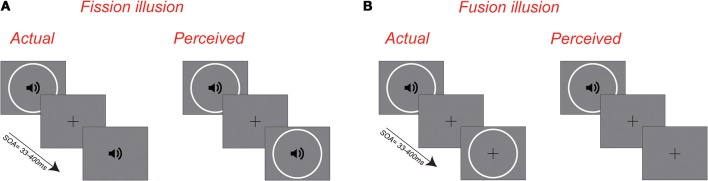
**Schematic illustration of the sound-induced *fission* and *fusion* illusions**. **(A)** The fission illusion refers to incidents whereby a single visual flash accompanied by two auditory tones is perceived as two flashes. **(B)** The fusion illusion refers to incidents whereby two visual flashes accompanied by a single auditory tone are perceived as one flash.

Audiovisual illusions provide a useful means of assaying multisensory integration in human observers. The sound-induced double-flash illusion (Shams et al., 2000), for example, refers to instances whereby a single visual flash accompanied by two auditory tones is erroneously perceived as two flashes (see Figure 1A). Whereas younger adults perceive this *fission* illusion only when the time interval between tones is relatively short (Shams et al., 2002), older adults are susceptible to this illusion across a much wider range of temporal asynchronies (Setti et al., 2011; Stapleton et al., 2014), presumably owing to an enlarged temporal window of integration (e.g., Diederich et al., 2008). This finding suggests that, under certain conditions, the integration of incongruous audiovisual signals leads to an age-related cost in perception. We wondered whether this cost of integration was due to the specific conditions of this illusion or represented a more general phenomenon associated with combining audiovisual input. For instance, it may be that older adults are generally more prone to perceiving multisensory illusions across a wider range of temporal asynchronies, indicative of a general cost associated with multisensory integration. On the other hand, the extent of the temporal window of integration is known to vary with different stimuli and task demands (e.g., Vatakis and Spence, 2006; Stevenson and Wallace, 2013) and it may be that older adults display an enhanced susceptibility to some multisensory illusions, but not others.

A lesser-known variant of the sound-induced flash illusion exists, in which two visual flashes accompanied by a single auditory beep are perceived as a single flash (Andersen et al., 2004). This fusion effect (see Figure 1B) is assessed in an identical manner to the fission illusion and is observed across a similar range of stimulus onset asynchronies in younger participants (Apthorp et al., 2013). As such, it provides an excellent means for assessing whether the age-dependent cost for multisensory integration extends to other conditions that involve the combination of audiovisual signals. To test this possibility, 25 younger and older participants performed an audiovisual task in which they had to indicate whether they perceived one or two flashes, whilst ignoring irrelevant auditory tones. Conditions known to produce fission and fusion were randomly interleaved with unisensory and multisensory control conditions to allow us to determine measures of response bias and participant lapses.

To preview our results, we replicated the original finding by Setti et al. (2011) that older adults are susceptible to the sound-induced fission illusion over a much wider range of cross-modal stimulus onset asynchronies than younger adults. However, this was not the case for trials that induced the fusion illusion where the performance of older adults was very similar to the younger group. Specifically, both groups were susceptible to the illusion when stimuli were separated by a short interval, but responded accurately in conditions with moderate to long stimulus onset asynchronies. These results point to dissociable age-related effects of multisensory integration and suggest that caution is required in interpreting multisensory behavioral effects in older adults.

## Methods

### Participants

Twenty five younger (10 male, age range: 18–30 years, mean age: 24 years old) and 25 older participants (9 male, age range: 65–88 years, mean age: 71 years old) volunteered to take part in the study. The younger participants were recruited from the student population of Trinity College Dublin and were compensated with research credits for their time. Older volunteers were community-living adults recruited through advertisements in local newspapers and community groups and were compensated for their travel expenses. All participants were naive to the purposes of the study and provided written consent to participate.

We assessed all older adults across a range of sensory functions and also on cognitive ability in order to screen for cognitive impairment. Visual acuity in near and far ranges was measured in older participants using the SLOAN Two-Sided ETDRS Near Vision and the 4 m 200 Series Revised ETDRS charts (Precision Vision, La Salle, and Illinois, USA), respectively. Contrast sensitivity was estimated using the Pelli-Robson Contrast Sensitivity Test. All older participants showed normal or corrected-to-normal acuity and contrast sensitivity for their age. Hearing ability was assessed using a modified version of the Hughson-Westlake method via a Kamplex BA 25 screening audiometer. All participants included in the study displayed thresholds within the normal limits for their age. The Montreal Cognitive Assessment was also administered to all the older participants to screen for cognitive impairment. Participants who scored below a score of 24/30 were excluded from the study. The Trinity College School of Psychology ethics board approved all recruitment and experimental procedures, and the experiment protocol was conducted in accordance with the principles of the Declaration of Helsinki.

### Apparatus and stimuli

Stimulus generation and presentation were controlled by an Apple Mac Pro computer on a HP L1710 monitor at a refresh rate of 60 Hz and a spatial resolution of 1280 × 1024 pixels. Participants were positioned at a distance of 57 cm from the screen, with head position supported by a chin rest. Experimental testing was conducted in a darkened, windowless room.

Stimuli were created and displayed in Matlab version 7.14 (R2012a) using Psychtoolbox (Brainard, 1997; Pelli, 1997). The visual stimulus was a hard-edged annulus presented at maximum luminance and displayed for 17 ms. The inner and outer edges of the annulus stimulus extended 8.5 and 10° from the center of the screen, respectively. The auditory stimulus was a brief auditory tone with a frequency of 3.5 KHz, which was presented for 10 ms via Sennheisser HD 202 headphones at a sound pressure level of 65 dB.

### Procedure

On each trial, participants were presented with one or two visual flashes, accompanied by one, two or no auditory beeps. Thus, there were six conditions in total, representing all possible combinations of flashes and beeps. For convenience, we will subsequently refer to these conditions by an abbreviation, which relates to their veridical percept. For example, trials described as 2F1B refer to those where two flashes were accompanied by one beep. For all conditions, participants were required to report how many flashes they perceived and were instructed to ignore the auditory beeps, which were irrelevant to the task. Participants indicated their response with a key press. Conditions known to produce the fission (1F2B) and fusion (2F1B) illusions were randomly interleaved with unisensory (1F and 2F) and multisensory (1F1B and 2F2B) control trials, such that there were six different conditions comprising an equal number of trials.

In conditions containing two flashes or two beeps, auditory and visual stimuli were separated by different stimulus onset asynchronies (SOAs). Seventeen younger and 25 older participants completed the experiment with 6 SOAs ranging between 33 and 400 ms (33, 50, 100, 150, 200, 400 ms), while 8 younger participants completed the experiment with 4 SOAs (50–200 ms). Participants collected a minimum of 10 trials per SOA for each condition, leading to total number of 360 trials (6 SOAs × 6 conditions × 10 repeats). At regular intervals over the course of the experiment, participants were prompted to take a self-timed break to avoid fatigue. The experiment lasted approximately 25–30 min.

### Data analysis

To examine the temporal bounds of susceptibility to the sound-induced flash illusion, we first examined the proportion of incorrect responses across all SOAs in the 1F2B and 2F1B conditions, known to produce the fission and fusion illusions, respectively. In a second analysis, we used signal detection theory to determine whether the change in the proportion of illusory reports resulted from changes in perceptual sensitivity, response bias, or both. This approach was based on that of Rosenthal et al. (2009) and further details of the analysis can be found there. Briefly, for each participant, we calculated the sensitivity (*d*′) and response bias (*c*) for discriminating between one and two flashes at each SOA and for each auditory beep condition separately. Perceptual sensitivity was calculated with the following equation:

(1) $$d^{\prime }=z(\text{H})-z(\text{FA})$$

where H denotes the proportion of correctly reported multiple flashes (i.e., hits), FA denotes the proportion of incorrectly reported multiple flashes, and *z*(p) represents the inverse of the cumulative normal distribution. Using these same definitions, response bias was calculated as:

(2) $$c=0.5{\;}^{*}(z(\text{H})\text{+z}(\text{FA}))$$

In cases where *p* = 0 or *p* = 1 (i.e., where participants reported all hits or no false alarms), these variables were approximated by 1/n and 1-1/n, respectively (where n is the total number of trials used to calculate H and FA).

Statistical analyses were conducted using mixed-model ANOVAs to analyse the effects of age group and SOA on the proportion of illusory responses, *d*′ and *c*. When appropriate, the Greenhouse-Geisser correction was applied to adjust the degrees of freedom of within-subject tests to correct for violations of the sphericity assumption and, in these cases, the adjusted *p*-value is reported. When multiple one-sample *t*-tests or pairwise comparisons were performed, the Bonferroni correction was used to maintain a family-wise Type I error rate at 0.05 and the adjusted *p*-value is reported.

## Results

**Figure 2:**
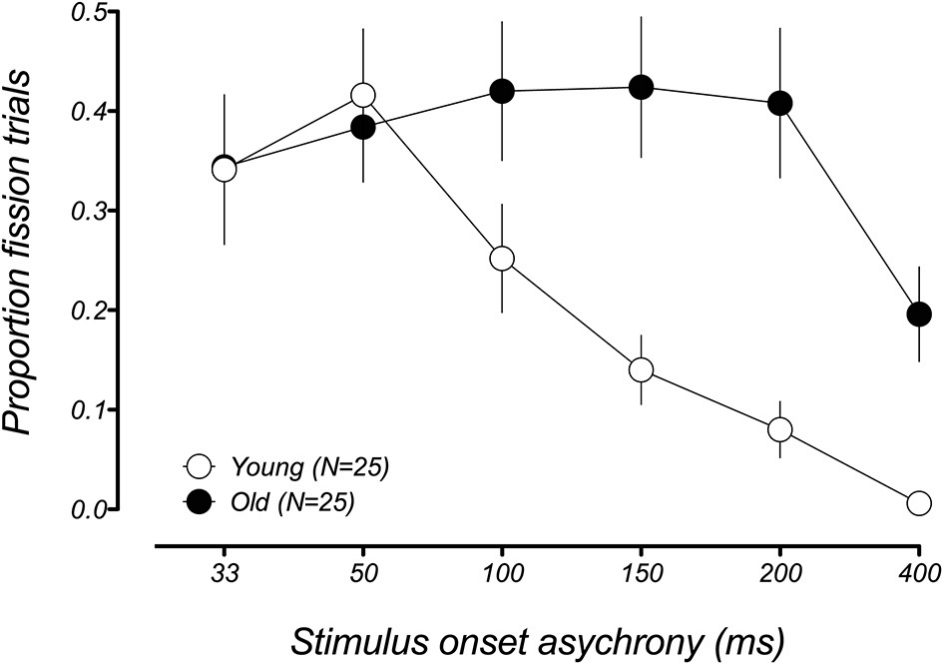
**Mean proportion of illusory responses in the fission condition (1F2B) as a function of stimulus onset asynchrony (SOA) for younger (white data points) and older (black data points) groups**. Both age groups experience the fission illusion on a similar amount of trials for short SOAs. However, whereas the illusion becomes less frequent for younger adults at longer SOAs, older adults still report the illusion on a significant proportion of trials for the longest SOA. Error bars represent ±1 *SE* of the mean.

We first compared the performance between younger and older groups in the conditions known to produce the fission illusion (1F2B). Figure 2 shows the group-averaged proportion of illusory responses to the 1F2B trials for both groups as a function of SOA. Young participants experienced the sound-induced fission illusion when the auditory beep stimuli were separated by short intervals, but their performance improved with increasing SOA, consistent with previous reports (Shams et al., 2002; Setti et al., 2011; Apthorp et al., 2013). In contrast, whereas older adults were as susceptible to the illusion as younger adults at shorter SOAs (33–50 ms), they remained susceptible to the illusion even for the longest SOA presented between the auditory beeps. A mixed-model 2 (age group) × 6 (SOA) ANOVA on performance to the 1F2B trials revealed significant main effects of SOA [*F*_(5, 200)_ = 8.92, *p*_adj_ < 0.001, GGeps = 0.48] and age group [*F*_(1, 40)_ = 8.09, *p* = 0.007], as well as a significant age group x SOA interaction [*F*_(5, 200)_ = 5.19, *p*_adj_ = 0.005, GGeps = 0.48], indicating that the temporal limits of the fission illusion differed in younger and older groups. To determine the range of SOAs producing the illusion in each age group, we compared the proportion of illusory responses at each SOA with veridical performance (i.e., error rate = 0) using one-sample *t*-tests. The younger group showed a significant fission effect for SOAs ranging between 33 and 150 ms [*ts*_(24)_ > 4.04, *p*_adj_ < 0.002] only, and not for longer SOAs [200 ms: *t*_(24)_ = 2.83, *p*_adj_ = 0.06; 400 ms: *t*_(16)_ = 1.0, *p*_adj_ = 1.0]. In contrast, the older group showed a significant fission effect at all six SOAs [33–400 ms, *ts*_(24)_ > 4.13, *p*_adj_ < 0.002].

This result replicates a previous finding from Setti et al. (2011), who reported that older adults were more susceptible to the sound-induced fission illusion across a wide range of SOAs compared to younger adults. One difference in the current study was the inclusion of the 400 ms condition. This was in part motivated by the fact that it was not clear from the Setti et al. study what interval between the auditory stimuli would be required to facilitate a return to veridical performance in older adults. Although there was a significant fission effect at 400 ms in older adults tested in the current study, it is clear that the illusion occurs less frequently than at shorter SOAs and a return to veridical performance at a longer duration appears likely. Coupled with the similarities in performance to the younger group at short SOAs, this improved performance at longer SOAs suggests that these effects may arise from an enlarged temporal window of integration of multisensory inputs in older adults (e.g., Diederich et al., 2008), a point we return to in the discussion.

**Figure 3:**
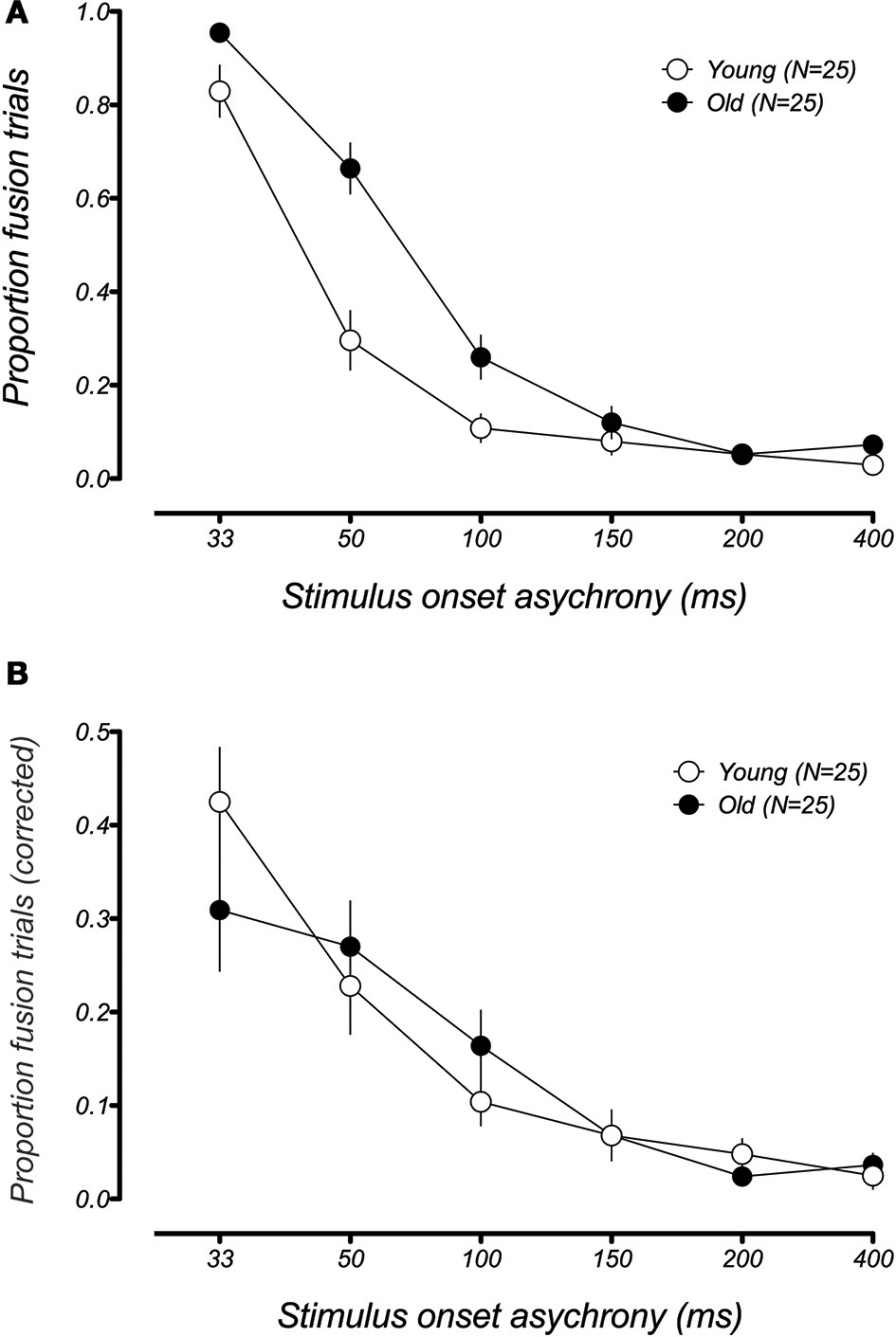
**Mean proportion of illusory responses in the fusion condition (2F1B) as a function of SOA for younger (white data points) and older (black data points) groups**. **(A)** Younger and older groups display similar performance on fusion trials, with both groups showing susceptibility to the illusion at short SOAs and a rapid decline in the illusion as the interval between flashes is increased. **(B)** Mean proportion of illusory fusion responses from **(A)** after being normalized by individual performance in the unisensory 2F condition. When individual and group differences in performance in the unisensory condition are taken into account in this way, the curves for both groups overlap each other, demonstrating that the temporal bounds of the fusion illusion does not differ across age groups. Error bars represent ±1 *SE* of the mean.

A very different pattern of results was observed in the 2F1B condition, which is known to produce the fusion illusion (Figure 3A). Similar to the fission illusion, the performance of the younger participants suggested a large fusion effect at short SOAs, while incidents of the illusion were relatively rare at longer intervals. While older adults appear to be more susceptible to the illusion at shorter SOAs, the temporal constraints of the effect were very similar to their younger counterparts. The higher proportion of illusion responses in the older group most likely reflects group differences in the unisensory conditions containing two flashes. Indeed, a paired sample *t*-test revealed that older participants were significantly less accurate than younger adults in the 2F condition at short SOAs [at 50 ms the proportion correct in younger and older groups was 0.91 and 0.64, respectively, *t*_(24)_ = 3.5527, *p* = 0.002]. This result is consistent with declines in temporal acuity with age (e.g., Misiak, 1951). Since the primary interest of the current study was in multisensory interactions, each participant's data were normalized by their accuracy level in the 2F condition at each SOA, to better reflect the proportion of fusion reports that occur as a result of the auditory tone rather than poor visual temporal resolution. Figure 3B shows the data replotted from Figure 3A, following this baseline correction. Represented this way, the data from the younger and older groups are almost identical and there was no significant effect of age group [*F*_(1, 40)_ = 0.048, *p* = 0.83], and no interaction between age group and SOA [*F*_(5,200)_ = 1.26, *p*_adj_ = 0.29, GGeps = 0.57], indicating that the magnitude and temporal limits of the illusion were very similar across younger and older adults. Moreover, comparing the normalized proportions of illusory responses with veridical performance at each SOA revealed that both younger and older participants experience the illusion for the same range of SOAs, namely 33–100 ms [younger: *t*_(24)_*s* >4.08, *p*_adj_ < 0.003; older *t*_(24)_*s* > 4.33, *p*_adj_ < 0.002], while performance did not differ from veridical performance for SOAs of 150 ms and longer [younger 150 ms: *t*_(24)_ = 2.53, *p*_adj_ = 0.11; older 150 ms: *t*_(24)_ = 2.65, *p*_adj_ = 0.08].

**Figure 4:**
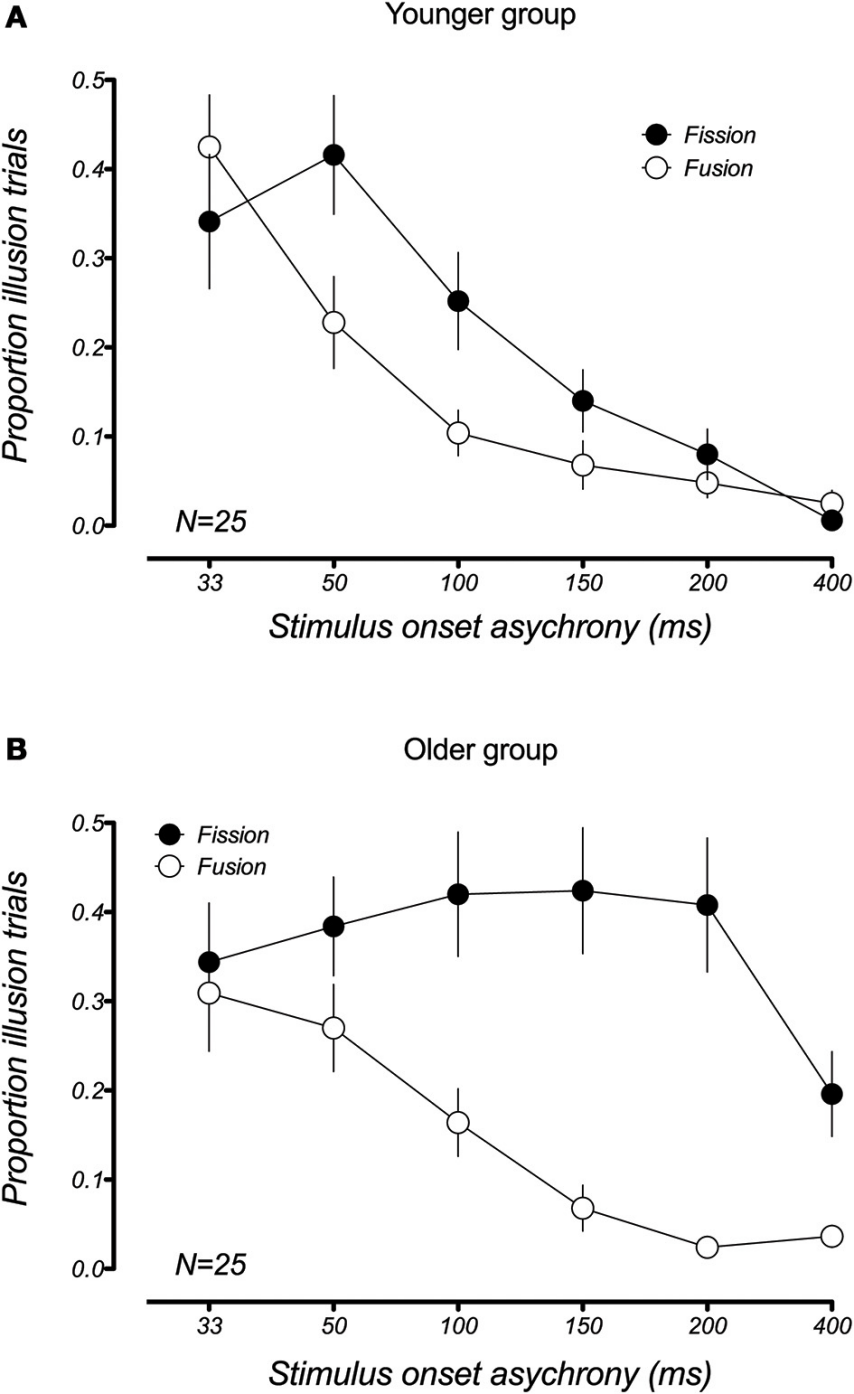
**Comparison of the mean proportion of illusory responses in the fission (1F2B) and fusion (2F1B) conditions within each age group**. **(A)** Younger adults showed a similar pattern of results for the fission and fusion illusions. **(B)** This is not the case in older adults, who show very different temporal constraints for the two illusions. Error bars represent ±1 *SE* of the mean.

Although the fission and fusion variants of the sound-induced flash illusion appear similar from a behavioral perspective, there is debate as to whether the two illusions are driven by the same or different neural processes (Mishra et al., 2007, 2008; Apthorp et al., 2013). Apthorp et al. suggested that two illusions stemmed from a common mechanism based on the high degree of similarity between the time courses of the fission and fusion illusion in younger participants. Consistent with this finding, our data also show this similarity in the temporal bounds of the illusions in younger participants (Figure 4A) and a 2 (illusion type) × 6 (SOA) repeated measures ANOVA confirmed that there was no significant main effect of illusion type [*F*_(1, 16)_ = 0.07, *p* = 0.79), and no significant illusion × SOA interaction [*F*_(5, 75)_ = 1.84, *p*_adj_ = 0.16, GGeps = 0.53]. However, this was clearly not the case for older participants (Figure 4B), where the fission and fusion effects had very different temporal constraints. For the older group, the ANOVA revealed a significant main effect of illusion type [*F*_(1, 24)_ = 19.5, *p* < 0.001], as well as a significant illusion x SOA interaction [*F*_(5, 120)_ = 6.16, *p*_adj_ = 0.001, GGeps = 0.54]. This difference in the temporal constraints of the two illusions in older adults supports the hypothesis that the two illusions result from distinct neural mechanisms.

**Figure 5:**
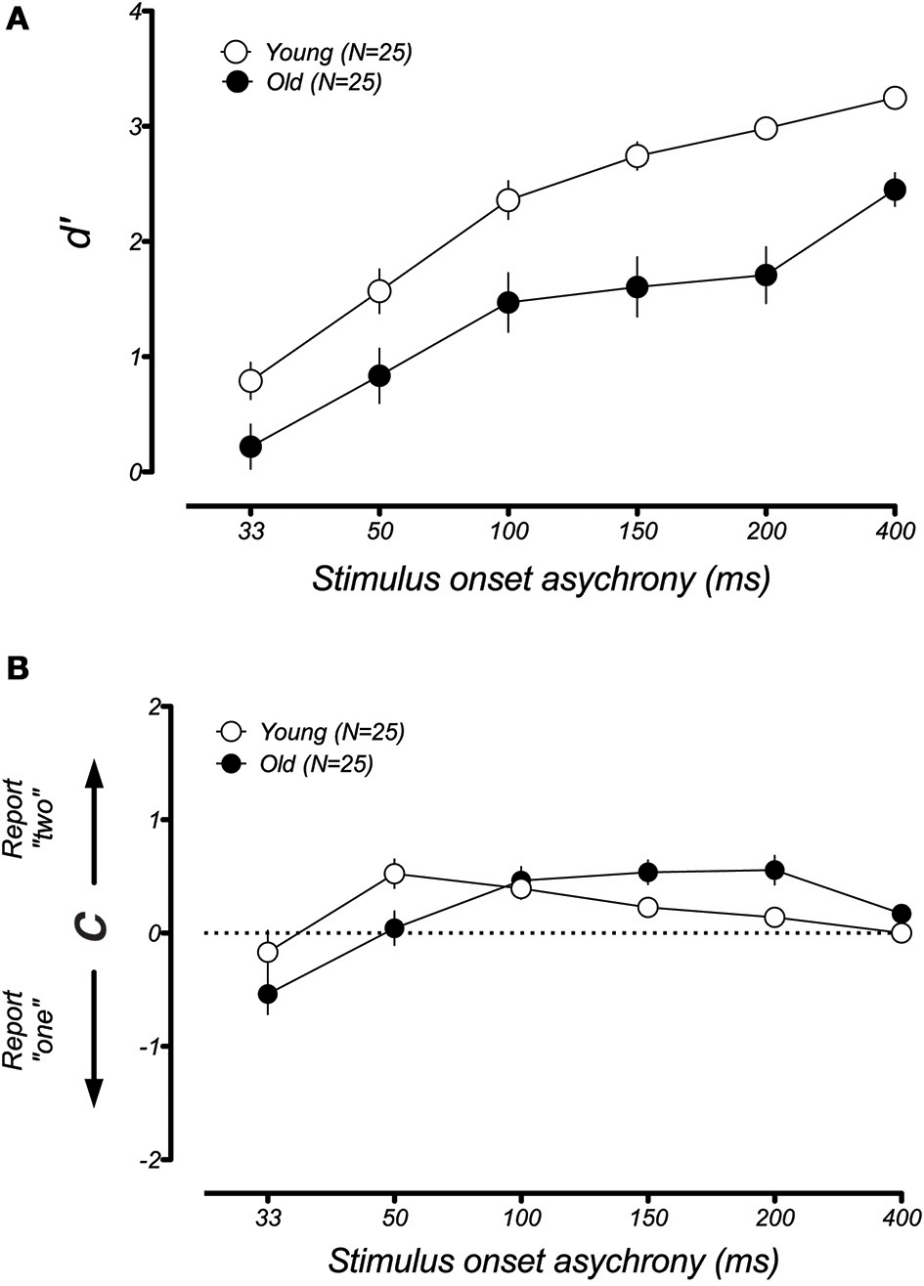
**Mean estimates of perceptual sensitivity *(d*′) and response bias *(c)* as a function of SOA for younger and older groups in the fission illusion**. **(A)** Both younger and older groups show similar trends with low sensitivity at short SOAs, but increases in discriminability at longer time intervals. However, the older group showed lower *d* ′ than the younger group across all SOAs. **(B)** In comparison, there was no systematic difference between the age groups in estimates of response bias, suggesting that age-related changes in perceptual sensitivity account for the increased susceptibility to the fission illusion. Error bars represent ±1 *SE* of the mean.

**Figure 6:**
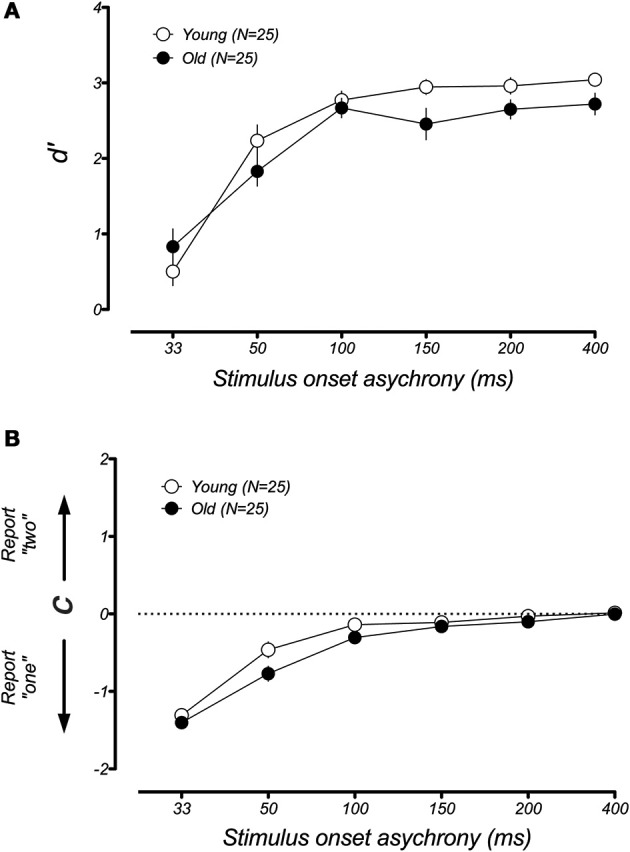
**Mean estimates of perceptual sensitivity *(d*′) and response bias *(c)* as a function of SOA for younger and older groups in the fusion illusion**. There was no significant difference of age group in measures of sensitivity **(A)** or response bias **(B)**. Error bars represent ±1 *SE* of the mean.

In younger participants, the sound-induced fission illusion has been shown to result from both a decrease in visual sensitivity and a shift in criterion (McCormick and Mamassian, 2008). We wondered which of these changes could explain the large performance difference between the younger and older groups in the fission condition. For instance, it could be that aging causes a genuine change in the perception of the visual flash during the trials that lead to the illusion. Alternatively, older participants might simply become confused or distracted by the presence of the auditory tone (e.g., Andres et al., 2006), leading to a larger response bias. To address this issue, we used a signal detection analysis to separate the changes in participant sensitivity from general shifts in response bias (see *Methods* and Rosenthal et al., 2009). Figure 5 plots the changes in *d*′ and response bias as a function of SOA for the fission illusion. For both groups, discrimination was poor at short SOAs and improved when the time interval between stimuli was made longer. However, a lower *d*′ was found for the older group than the younger group across all SOAs and there was a significant difference between the age groups [*F*_(1, 40)_ = 19.49, *p* < 0.001]. In contrast, estimates of response bias were similar across both young and old groups and there was no significant group effect [*F*_(1, 40)_ = 0.34, *p* = 0.53]. Thus, it appears that the age-related difference in performance in the sound-induced fission illusion resulted from a reduction in perceptual sensitivity of older adults. In the case of the fusion illusion (Figure 6), there was no significant differences between the age groups for measures of *d*′ [*F*_(1, 40)_ = 0.97, *p* = 0.33] or response bias [*F*_(1, 40)_ = 2.41, *p* = 0.13].

**Figure 7:**
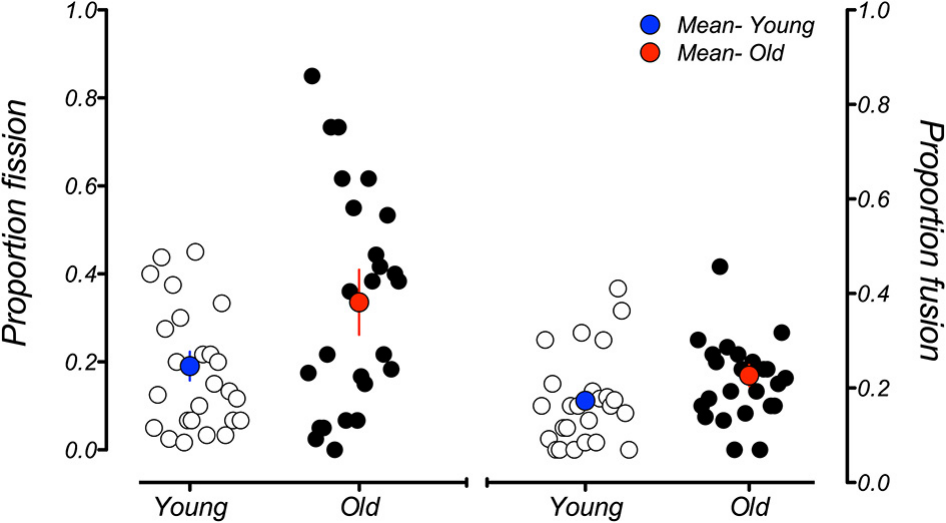
**Mean proportion of illusory responses across all SOAs for individual participants in the younger (white data points) and older (black data points) groups for the fission illusion (left-hand side) and fusion illusion (right-hand side)**. Blue and red data points represent the mean susceptibility of the younger and older groups, respectively. While there is some variability in susceptibility to the fission illusion in the younger group, this variability is much greater in older participants. Individual differences in the fusion illusion are approximately the same across age groups. Error bars represent ±1 *SE* of the mean.

A commonly reported observation regarding the sound-induced fission illusion is its high degree of between-subject variability (e.g., Mishra et al., 2007; Stevenson et al., 2011; de Haas et al., 2012). We also observed individual differences of this nature and were interested in how much it could account for the age-related differences we see in the group-averaged data. Figure 7 plots each individual's performance, averaged across all SOAs, for the fission and fusion illusions. Plotted this way it is clear that, while individual differences do exist for the fission illusion in younger adults, the degree of variability is much higher in older adults. The data also suggest that there may exist two distinct groups within the current older cohort: those whose performance is located below the group mean data point, who experienced the fission illusion to a similar extent to the younger group, and those whose performance is above the mean data point, who were much more susceptible to the fission illusion. Importantly, this difference cannot be explained by an effect of aging within our older sample. When the magnitude of individual fission effects in older participants were plotted as function of participant age, there was a modest positive slope in the linear regression line fitted through the data (0.011) indicating some relationship between these variables. However, the slope did not significantly differ from zero (*p* = 0.192), suggesting that this split in the older group is not solely due to age of the participants. The reasons for the difference between these individuals are not clear, but ongoing work in our laboratory is attempting to understand the factors that lead to this variability in performance across participants. In contrast, the variation in performance in the fusion conditions was much smaller and there were no obvious differences between the young and old groups.

## Discussion

The aging process leads to significant changes in all sensory systems and a variety of cognitive functions. Multisensory integration plays a key role in bridging the gap between these sensory functions and higher-order cognitive processing, yet research into the effects of aging on this process has been equivocal (Laurienti et al., 2006; Poliakoff et al., 2006; Peiffer et al., 2007; Setti et al., 2011, for a review see Mozolic et al., 2012). In the current study, we replicated previous findings showing that older adults are susceptible to the sound-induced *fission* illusion across a wider range of SOAs than younger adults (e.g., Setti et al., 2011). We extended this line of research by showing that this enhanced susceptibility to the illusion results from changes in perceptual sensitivity, rather than changes in response bias, and that the older group was significantly more variable in their susceptibility to the fission illusion than their younger counterparts. Surprisingly, however, we did not observe equivalent age-related changes in susceptibility to the sound-induced *fusion* illusion, with older adults performing on a par with the younger group. In the following section, we discuss the role of cognitive factors on our results and suggest potential mechanisms to explain the discrepancy in the susceptibility of older adults to the fission and fusion illusions.

Multisensory integration plays an important intermediary role between perception and cognition, where the brain must merge bottom-up, stimulus-driven input from primary sensory areas with top-down guidance from a range of cognitive processes. For example, integration of perceptual signals across the senses can capture attention through bottom-up processes, while top-down selective attention can facilitate the integration of multisensory inputs or lead to a spread of attention across the senses depending on the particular task demands (e.g., Talsma and Woldorff, 2005; Talsma et al., 2010). As such, the role of cognitive processes, such as top-down attention, must be considered in explaining any multisensory effect. This task becomes considerably more difficult when assessing multisensory perception in older adults, given the systematic and age-dependent decreases in unisensory function, as well as changes in selective attention that are known to influence both unisensory (e.g., Hasher et al., 1991; Alain and Woods, 1999) and cross-modal perception (e.g., Andres et al., 2006; Poliakoff et al., 2006). For example, some studies show that older adults are more likely to become distracted by irrelevant auditory information when performing an auditory-visual oddball task (Andres et al., 2006), suggesting an impaired ability to filter out task-irrelevant auditory noise. However, other evidence suggests that older adults are not impaired in their ability to selectively attend to the visual modality (Hugenschmidt et al., 2009).

Could age differences in attentional control explain the increased susceptibility to the sound-induced fission illusion experienced by older adults? We suspect not for two reasons. First, one might expect that any increase in distractibility would lead to an increase in response bias, rather than affecting perceptual sensitivity, as the participant would be tempted to respond in line with the number of auditory stimuli presented. However, our signal detection analysis revealed that age-related increases in susceptibility to the illusion were primarily due to decreases in sensitivity (*d*′), indicating that the auditory beeps generated illusory second flashes (see also McCormick and Mamassian, 2008). Second, if a deficit in selective attention underpinned the increased susceptibility to the fission illusion, we would expect to observe a similar pattern of results for the fusion illusion, given the similarities in the task structure and experimental conditions. This was not the case, however, with the younger and older groups showing similar performance in fusion conditions. Thus, it is unlikely that the current results can be explained by age-related deficits in suppressing irrelevant auditory stimuli. This is not to fully rule out the influence of cognitive factors on our results, however, which undoubtedly play a role. In particular it seems likely that differences in cognitive function might help to explain the increased inter-subject variability in the older group performance on the fission illusion. This is a hypothesis we are currently pursuing.

The current data show that while older adults are susceptible to the fission illusion across a wider range of SOAs than their younger counterparts, their performance does appear to recover if the interval between the auditory tones is long enough. Similar to findings in other studies of multisensory processing in older adults (Laurienti et al., 2006; Peiffer et al., 2007; Diederich et al., 2008), this pattern of results is consistent with the notion of an extended temporal window of integration in older adults. This broader temporal window is believed to arise from slowing of peripheral sensory processing, rather than general cognitive decline (Diederich et al., 2008), and has been proposed to help compensate for unisensory deficits in certain conditions (Laurienti et al., 2006; Peiffer et al., 2007). Based on these findings, we expected older adults would also be susceptible to the fusion illusion across a larger range of SOAs than younger participants. This did not prove to be the case, however, with older adults displaying a similar level of performance to the younger group.

What could cause these large differences in age-related susceptibility to the fission and fusion illusions? One possible explanation is that the fusion illusion does not constitute a genuine example of multisensory integration. This explanation seems unlikely, however. Both EEG and fMRI studies (Watkins et al., 2007; Mishra et al., 2008) investigating the neural correlates of the fusion illusion have demonstrated a causal role between activity in the superior temporal sulcus, a brain region believed to play a pivotal role in multisensory integration (Beauchamp et al., 2004), and the subjective experience of the illusion. For instance, Watkins et al. (2007) compared trials in which participants experienced the fusion illusion with those where they did not. In trials where participants reported the illusion, there was increased BOLD activity in the right superior temporal sulcus and decreased activity in primary visual cortex and this was not the case when participants responded veridically. This pattern of results is consistent with the idea that the subjective experience of the illusion is mediated by feedback connections from polymodal areas to primary sensory cortices (Mishra et al., 2008) and suggests that the sound-induced fusion illusion is a *bona fide* example of multisensory integration.

A more likely explanation for this discrepancy between age-related fission and fusion effects is that each variant of the illusion has its own temporal integration window derived from distinct networks of activation in the brain (Mishra et al., 2007, 2008). This explanation is consistent with previous research showing large differences in the size of temporal integration windows depending on the type of stimulus to be integrated and the task to be performed (Vatakis and Spence, 2006; Stevenson and Wallace, 2013). It is also consistent with EEG studies that show significant differences in the timing and localization of the major ERP components associated with each illusion (Mishra et al., 2007, 2008). In these studies, Mishra et al. combined an ERP difference analysis with source localization techniques to identify the different patterns of cortical activity underlying each variant of the illusion. A trial-by-trial analysis of ERPs suggested that the latencies of the major components underlying the fission illusion occurred at 110, 120, and 130 ms, reflecting activity in auditory, visual and superior temporal cortices, respectively (Mishra et al., 2007). On the other hand, the major components for the fusion illusion were observed at much later latencies (180 and 240 ms), but again involved feedback from superior temporal sulcus to visual cortex (Mishra et al., 2008). Interestingly, the overall pattern of cortical activity for each illusion differed markedly from that of the congruent audiovisual input (i.e., 1F1B or 2F2B). From these findings the authors concluded that, despite appearing to be reciprocal perceptual phenomena, very different neural circuits underlie the fission and fusion illusions.

These findings may help to explain the dissociation between the older group's performance on the fission and fusion illusions in the current study. For instance, it is conceivable that the cortical network underlying the fusion illusion could be preserved in older adults, while one or more areas within the fission network may experience a decrease in processing speed. Indeed, Peiffer et al. (2009) found that the pattern of cross-modal deactivation of the visual cortex by auditory stimuli differed between younger and older adults, indicating age-related changes in cross-modal interactions between sensory cortices. Importantly, the current results, together with those from Mishra et al. (2007, 2008), suggest that a degree of caution is required in interpreting how multisensory integration is affected by age on the basis of a single behavioral effect. Rather, these findings suggest that a more prudent approach would be to treat each study independently, considering factors such as the particular task demands, the quality and type of sensory inputs involved, as well as the underlying neural mechanisms that give rise to the effect in question.

### Conflict of interest statement

The authors declare that the research was conducted in the absence of any commercial or financial relationships that could be construed as a potential conflict of interest.
